# Comparison of tracer kinetic models for ^68^Ga-PSMA-11 PET in intermediate-risk primary prostate cancer patients

**DOI:** 10.1186/s13550-023-01066-2

**Published:** 2024-01-10

**Authors:** Nathaniel J. Smith, Mark A. Green, Clinton D. Bahler, Mark Tann, Wendy Territo, Anne M. Smith, Gary D. Hutchins

**Affiliations:** 1 Indiana University School of Medicine, 950 West Walnut Street, Indianapolis, IN 46202 USA; 2 Weldon School of Biomedical Engineering, Purdue University, West Lafayette, IN USA; 3 Siemens Medical Solutions USA, Inc., Knoxville, TN USA

**Keywords:** ^68^Ga-PSMA-11 PET, Tracer kinetic model, Compartmental model, Graphical model, Patlak analysis, Primary prostate cancer, Dynamic imaging

## Abstract

**Background:**

^68^Ga-PSMA-11 positron emission tomography enables the detection of primary, recurrent, and metastatic prostate cancer. Regional radiopharmaceutical uptake is generally evaluated in static images and quantified as standard uptake values (SUVs) for clinical decision-making. However, analysis of dynamic images characterizing both tracer uptake and pharmacokinetics may offer added insights into the underlying tissue pathophysiology. This study was undertaken to evaluate the suitability of various kinetic models for ^68^Ga-PSMA-11 PET analysis. Twenty-three lesions in 18 patients were included in a retrospective kinetic evaluation of 55-min dynamic ^68^Ga-PSMA-11 pre-prostatectomy PET scans from patients with biopsy-demonstrated intermediate- to high-risk prostate cancer. Three kinetic models—a reversible one-tissue compartment model, an irreversible two-tissue compartment model, and a reversible two-tissue compartment model, were evaluated for their goodness of fit to lesion and normal reference prostate time-activity curves. Kinetic parameters obtained through graphical analysis and tracer kinetic modeling techniques were compared for reference prostate tissue and lesion regions of interest.

**Results:**

Supported by goodness of fit and information loss criteria, the irreversible two-tissue compartment model optimally fit the time-activity curves. Lesions exhibited significant differences in kinetic rate constants (*K*_1_, *k*_2_, *k*_3_, *K*_i_) and semiquantitative measures (SUV and %ID/kg) when compared with reference prostatic tissue. The two-tissue irreversible tracer kinetic model was consistently appropriate across prostatic zones.

**Conclusions:**

An irreversible tracer kinetic model is appropriate for dynamic analysis of ^68^Ga-PSMA-11 PET images. Kinetic parameters estimated by Patlak graphical analysis or full compartmental analysis can distinguish tumor from normal prostate tissue.

**Supplementary Information:**

The online version contains supplementary material available at 10.1186/s13550-023-01066-2.

## Background

Prostate cancer has an estimated lifetime incidence of 1 in every 9 men, but it is estimated that between 20 and 40% of serum PSA-motivated prostate cancer evaluations reflect low-grade, non-malignant changes [1, 2]. Surgery and radiotherapy significantly reduce the prevalence of metastatic disease progression, but may also cause erectile dysfunction and/or urinary incontinence [3]. Appropriately specific diagnostics can reduce the incidence of overtreatment and improve patient-specific outcomes. Positron emission tomography (PET) imaging with the urea-based prostate-specific membrane antigen (PSMA) targeted ^68^Ga-Glu-NH-CO-Lys-(Ahx)-HBED-CC (^68^Ga-PSMA-11) has greatly improved the diagnosis and treatment planning for prostate cancer, as upregulated PSMA expression has been linked with aggressive or advanced disease [4, 5].

The ^68^Ga-PSMA-11 tracer standardized uptake value (SUV) correlates with pathological Gleason grade and can support surgical planning as well as detect nodal metastases and biochemical recurrence [6, 7]. SUVs are commonly favored for their ease of clinical implementation, but SUVs depend on accurate dose and scanner cross-calibration, the time between injection and imaging, image acquisition characteristics (scanner, scatter/attenuation correction, reconstruction, frame duration), patient weight and radiopharmaceutical distribution characteristics, and may be affected by patient motion or partial volume effects. Additionally, ^68^Ga-PSMA-11 does not accumulate in muscle or adipose tissue, and SUV body mass normalization may increase the variability of uptake calculations [8]. Differences in acquisition and reconstruction parameters can also make the comparison of SUVs across different patients and acquisition timepoints error-prone, especially when numerical cutoffs are used [9].

Kinetic modeling of tracer binding interactions reduces the impact of errors associated with patient weight, uptake timing, and dose calibration [10]. Unlike SUV analysis and simple static images, dynamic PET imaging with ^68^Ga-PSMA-11 may be used to distinguish physiologic differences in receptor-ligand affinity, receptor availability, and ligand delivery and extraction, which are considered in aggregate with SUV analysis [11]. These physiologic parameters provide additional information which can improve tissue characterization [12, 13]. However, few studies have compared compartmental models for ^68^Ga-PSMA-11, and there is not a clear consensus for whether a reversible or irreversible two-tissue compartmental model optimally suits ^68^Ga-PSMA-11 PET data [14, 15].

^68^Ga-PSMA-11 is rapidly cleared from the blood, and blood metabolite components may be assumed negligible for the compartmental model [16]. This study aimed to verify the findings by Ringheim et al. and confirm the use of an irreversible two-tissue compartment model for ^68^Ga-PSMA-11 PET analysis [15].

## Methods

### Patients

Eighteen men with a total of 23 lesions were included in this retrospective evaluation (NCT04936334), after two patients were removed from the study cohort due to excessive motion during imaging. This study was approved by the institutional review board, and informed consent was obtained for all individuals prior to imaging. Men with histologically-proven prostate cancer who were scheduled for prostatectomy were eligible for this study if they were over the age of 18 and had at least NCCN intermediate-risk disease or 3 cores of at least Gleason 3 + 4 disease. Patients needed to be able to lay still for the entire 60-min PET/CT scan and were excluded if they had received treatments with ionizing radiation within the past 30 days. Following prostatectomy, all prostates were analyzed as whole-mount pathological specimens by a board-certified pathologist. Whole-mount pathological findings served as the reference standard for regional tissue classification. The study patients had elevated serum PSA values (median 6.8, range 4.1–20.6 ng/mL) and enlarged prostates (median 40.4 mL, range 27.3–89.4 mL) and were primarily white (17/18). The median patient age was 65 (range 52–75), and the median patient body weight was 90.7 kg (range 63.5–132.0 kg). A more complete charting of patient demographics is contained in Table 1.

**Table 1:** Patient characteristics, injected doses, and summary pathology classification

Subject	Age	Prostate volume (cc)	PSA (ng/mL)	Subject Weight (kg)	Injected dose (MBq)	Number of lesions	Lesion location (Gleason grade group)
1	69	35.2	9.6	108.4	183.2	1	RTZ (2), RCZ (1)
2	62	36.1	20.6	132.0	180.6	1	LPZ (3)
3	69	60	9.8	76.2	183.5	1	RCZ (4), RPZ (3)
4	69	39.1	5.6	97.5	183.2	1	LPZ (3)
5	63	30	4.2	79.4	181.3	1	LPZ (2)
6	57	28.5	5.6	90.7	170.6	1	RPZ (2)
7	52	27.3	6.2	111.1	171.7	2	RTZ (2), RPZ (2), LTZ (2)
8	57	51	10.8	108.0	182.4	2	LCZ (2), LTZ (2), RPZ (2)
9	64	43.8	4.8	83.9	185.0	1	RPZ (3)
10	66	59.4	18.5	88.5	185.0	1	LPZ (3)
11	53	37.9	9.2	91.2	183.9	1	LCZ (3)
12	69	66.5	8.7	63.5	184.6	1	LPZ (3)
13	60	41.6	4.5	92.1	186.1	1	RPZ (2)
14	75	37.4	6.6	81.7	184.6	1	LPZ (2)
15	58	62.8	6.7	112.0	185.7	1	RPZ (2)
16	66	41.9	6.8	90.7	183.2	2	LCZ (2), RPZ (1)
17	66	89.4	7.3	69.0	184.3	2	RTZ (2), RPZ (2)
18	67	72.6	4.1	88.0	183.5	2	RPZ (2)

PSA—prostate-specific antigen. The lesion locations are described as the anatomical left (L) or right (R) central zone (CZ), transitional zone (TZ), or peripheral zone (PZ) based on postsurgical whole-mount pathology

### PET/CT acquisition protocol

Patients received a 55-min dynamic PET scan acquired in list-mode, centered over the pelvis. Images were acquired with a Siemens Biograph Vision 600 Edge scanner (Siemens Healthineers, Knoxville, USA). The ^68^Ga-PSMA-11 radiopharmaceutical was prepared as previously reported [17, 18]. At the start of the PET scan, patients received a bolus injection of [^68^Ga]-PSMA-11 (median 183.5 MBq, range 170.6–186.1 MBq), followed by a 10 mL saline flush. PET images were reconstructed with a 3D ordered-subsets expectation maximization (OSEM) algorithm with point-spread function (PSF) and time of flight (TOF) [3i5s, 3.5 mm FWHM spatial resolution, 210 ps temporal resolution, 1.42 × 1.42 × 3.0 mm voxels, 5 mm Gaussian smoothing]. The images were corrected for decay, attenuation, scatter, dead time, random coincidences and were detector-normalized. The PET images were then processed into 40 temporal frames (12 × 5 s, 12 × 10 s, 6 × 20 s, 10 × 300 s).

Computed tomography (CT) images were acquired sequentially with the PET scan (120 kV peak, 330 ms exposure time, 658 mA tube current, 0.98 × 0.98 × 1.00 mm voxels, 500 mm field of view) using a soft tissue kernel (Br38).

### Image analysis

**Fig. 1:**
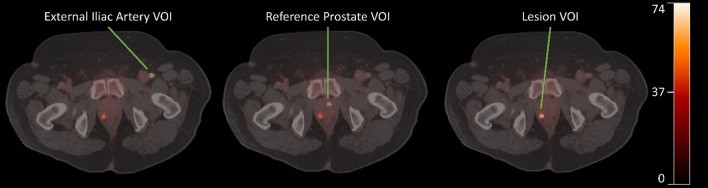
10 mm spherical VOI placements for artery (left), normal prostate tissue (middle), and lesion (right). An early bolus phase image was used to locate the external iliac artery. All three displayed images are 40–55 min PET frames overlaid with CT. Scale bar is in units of kBq/mL

The reconstructed PET/CT images were analyzed by a board-certified nuclear medicine physician and a board-certified urologist using in-house software (*Q-Image*) built using IDL (L3Harris Geospatial, Boulder, CO, USA). Forty cubic millimeter (~ 50 voxel) spherical reference regions of interest (ROIs) were sampled in the central, peripheral, and transitional prostatic zones in the left and right hemispheres. Separate ROIs were also contoured under physician guidance for the index lesion, contralateral reference region, and secondary lesions when present, as shown in Fig. 1. Accuracy of PET region placements in reference prostate and lesion was retrospectively confirmed with post-surgical whole-mount pathology specimens interpreted by a board-certified pathologist. Time-activity curves (TACs) were extracted in Bq/mL units. SUVs were calculated using the final 15 min of the scan, and mean SUV was calculated for each ROI.

### Image-derived input function

The image-derived input function (IDIF) was calculated using in-house software built in IDL. A linear segment of the iliac artery (approximately 20.0 mm) was identified on a bolus phase PET image (approximately the first 60 s of data acquisition). Profiles across the vessel were generated at each location along the length of the vessel that fell within the boundaries of the iliac artery segment. Each profile was fit with a vessel profile model (vessel width step function convolved with scanner resolution kernel) to estimate the vessel diameters. The average vessel diameter was then used to generate a 3D vessel model with a background region that was large enough to capture all signal spillover into the vessel region. An arterial input function volume of interest (VOI) was then placed at the center of the vessel region to eliminate resolution distortions that would occur near the edges of the vessel segment. Simulated PET images were generated by convolving the 3D vessel and background regions individually, which were used to estimate the contribution of each region to the arterial input function VOI. The individual contributions represented the fraction of the arterial blood signal that contributed to the arterial input function VOI (F_A_) and the fraction of the background region signal that contributed to the arterial input function VOI (F_B_). The estimation of resolution distortion corrected arterial blood concentration C_A_(t) was calculated using the following equation for each image frame where C_VOI_(t) is the tracer concentration in the arterial input function VOI, C_T_(t) and V_T_ are the tracer concentration and volume of the combined artery and background region, V_B_ is the volume of the background region, and V_A_ is the volume of the arterial input function VOI.

$${C}_{A}\left(t\right)=\frac{\frac{{C}_{VOI}\left(t\right)}{{F}_{B}}- {C}_{T}\left(t\right)\frac{{V}_{T}}{{V}_{B}}}{\left[\frac{{F}_{A}}{{F}_{B}}-\frac{{V}_{A}}{{V}_{B}}\right]}$$

**Fig. 2:**
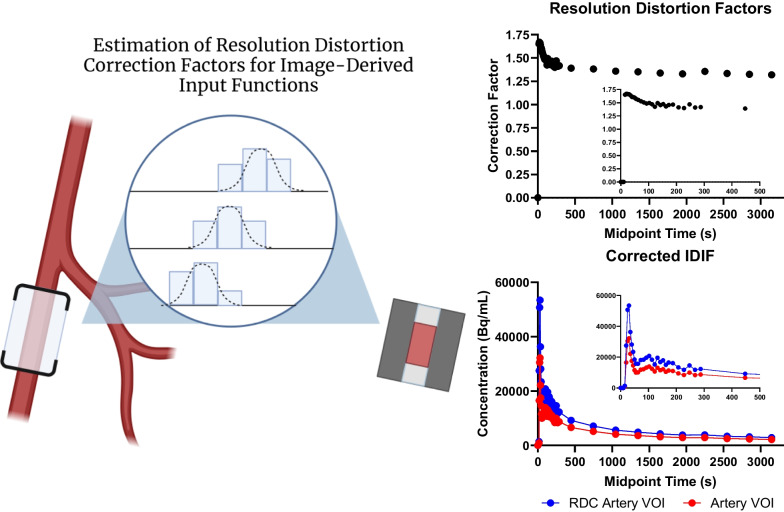
Representation of the resolution distortion correction method for estimating the IDIF. A 20 mm segment of the iliac artery was manually defined in a region with homogenous background signal. Profiles across the vessel were generated at each location along the length of the vessel segment. Each profile was fit with a vessel profile model to estimate the vessel diameters. The mean vessel diameter estimate was then used to generate a 3D vessel model. Simulated PET images were generated by convolving the 3D vessel and background model to estimate resolution distortion correction factors (shown in upper right for a single patient). Resolution-distortion corrected time-activity curves (lower right) for the vessel region were then generated using the distortion correction factors and original time-activity curves. IDIF—image-derived input function. RDC—resolution distortion corrected. VOI—volume of interest

An example of the resolution distortion correction algorithm is demonstrated in Fig. 2.

### Kinetic analysis and model validation

Three different kinetic models were evaluated in this analysis: a reversible one-tissue compartment model with two-rate constants (1T2k), an irreversible two-tissue compartment model with three-rate constants (2T3k), and a reversible two-tissue compartment model with four rate constants (2T4k). ^68^Ga-PSMA-11 was assumed to be uniformly distributed in plasma with no uptake into red blood cells. Whole blood was weighted by an assumed 38% hematocrit, and no metabolite components were included [15]. Model optimality was evaluated based on chi-square goodness-of-fit criteria and the Akaike information criterion (AIC), consistent with other studies [9]. The 2T3k model net influx rate ${K}_{i}={K}_{1}{k}_{3}/({k}_{2}+{k}_{3})$ and distribution volume ${V}_{d}={K}_{1}/({k}_{2}+{k}_{3})$ were evaluated from the full compartmental model as well as the Patlak graphical method [19]. A full tabulation of model parameters can be found in Table 2.

**Table 2:** Parameter definitions

Parameter	Definition	Calculation	Units
*K*_1_	Rate constant, transfer of tracer from plasma to first tissue compartment	Modeled	$\frac{{{\text{ml}}}_{{\text{blood}}}}{{{\text{ml}}}_{{\text{tissue}}}\cdot {\text{min}}}$
*k*_2_	Rate constant, transfer of tracer from first tissue compartment to plasma	Modeled	${{\text{min}}}^{-1}$
*k*_3_	Rate constant, transfer of tracer from first tissue compartment to second tissue compartment	Modeled	${{\text{min}}}^{-1}$
*k*_4_	Rate constant, transfer of tracer from second tissue compartment to first tissue compartment	Modeled	${{\text{min}}}^{-1}$
*K*_*i*_	Net tracer influx rate	${K}_{i}=\frac{{K}_{1}{k}_{3}}{({k}_{2}+{k}_{3})}$	$\frac{{{\text{ml}}}_{{\text{blood}}}}{{{\text{ml}}}_{{\text{tissue}}}\cdot {\text{min}}}$
*V*_*d*_	Distribution volume of tracer	${V}_{d}=\frac{{K}_{1}}{({k}_{2}+{k}_{3})}$	$\frac{{{\text{ml}}}_{{\text{blood}}}}{{{\text{ml}}}_{{\text{tissue}}}}$
SUV	Standardized uptake value	${\text{SUV}}=\frac{{C}_{{\text{PET}}}\left(t\right)}{{\text{Dose}}/{\text{BodyMass}}}$	$-$
%ID/kg	Percentage of total injected dose per kilogram of tissue	$\%{\text{ID}}/{\text{kg}}=\frac{{C}_{{\text{PET}}}\left(t\right)}{{\text{Dose}}}$	$-$

### Statistical analysis

Statistical tests were performed with GraphPad Prism 9.5.0 (GraphPad, San Diego, California, USA). Significance was set at 5%, and all variables are reported with median and range or mean and standard deviation. The distributions of all numerical variables were tested for normality. Kinetic and semiquantitative parameters were compared for lesion, and reference tissue regions using a patient-wise paired Šídák's test for multiple comparisons. Linear models and Pearson correlations were calculated to assess the association between compartmental parameters, Patlak graphical parameters, and SUVs. The kinetic models were compared for goodness of fit across the central, transitional, and peripheral prostate, and kinetic parameters were compared for consistency across the three prostate zones.

## Results

### Model selection

**Fig. 3:**
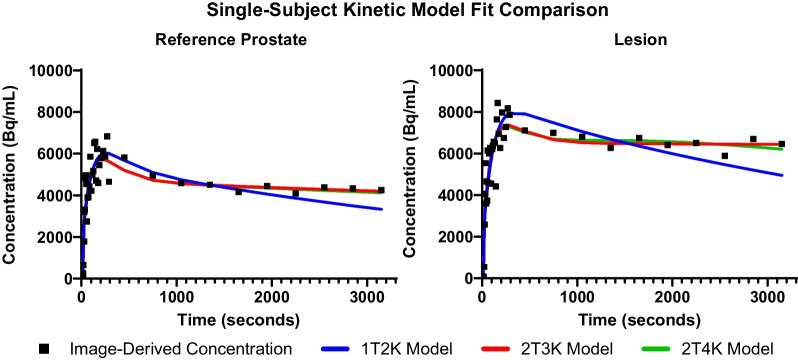
Comparison of three tracer kinetic model fits for the reference prostate and two lesions originating from subject 17. Model configurations include a one-tissue (1T) and two-tissue (2T) compartment model, with two, three, or four parameters (2K, 3K, 4K, respectively). SUV for the lesion was 3.0

**Fig. 4:**
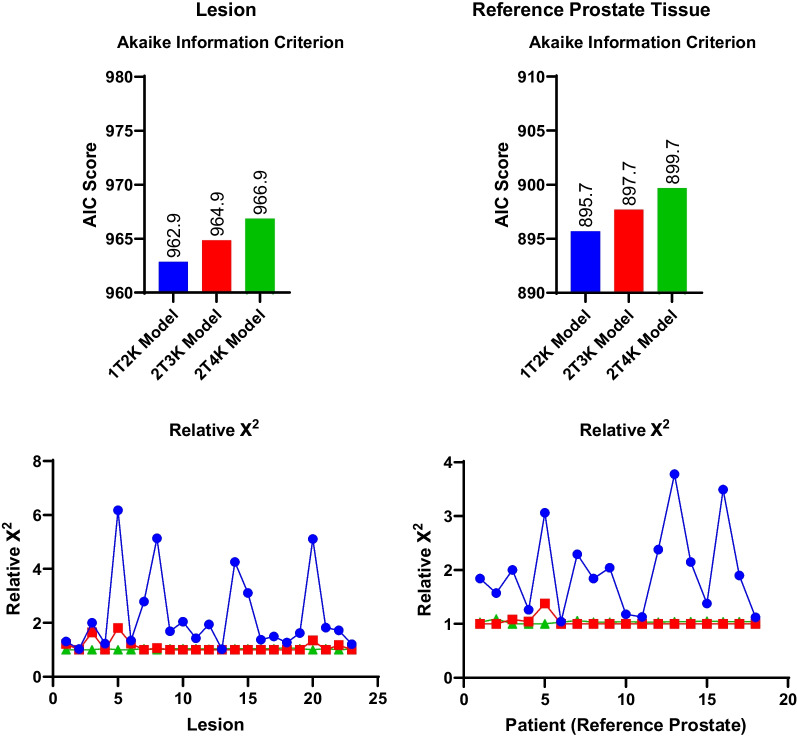
Model selection criteria for three ^68^Ga-PSMA-11 tracer kinetic models. Blue refers to the one-tissue compartment, two rate constant (1T2K) model. Red shows the two-tissue compartment, three parameter (2T3K) model, and green shows the two-tissue compartment, four parameter (2T4K) model. Relative ${\rm X}^{2}$ goodness-of-fit values are normalized to the model with the minimal ${\rm X}^{2}$ values for each patient

Example VOI placements for artery, reference prostate, and lesion are shown in Fig. 1. A sample extracted TAC and modeled curve fits are demonstrated for a lesion and reference prostate in Fig. 3. Chi-square goodness of fit and AIC values for the 1T2K, 2T3K, and 2T4K compartmental models are shown in Fig. 4. All three models performed similarly for prostate and reference tissue regions, with a $\mathrm{\Delta AIC}$ of 2.0 for the 2T3K model and a $\mathrm{\Delta AIC}$ of 4.0 for the 2T4K model, in reference to the 1T2K exchange model. Therefore, despite similar $\mathrm{\Delta AIC}$ criteria values between successively parametrized models, the 2T4K model did not adequately improve the TAC fit when corrected for additional parameter bias, relative to the 1T2K model according to rules established by Burnham and Anderson [20]. Relative ${\rm X}^{2}$ goodness-of-fit criteria support the use of the 2T3K and 2T4K models, as ${\rm X}^{2}$ is significantly reduced for the 2T3K (${\rm X}_{diff}^{2}=14.76$, $d{f}_{diff}=1$, $p<0.001$) and 2T4K (${\rm X}_{diff}^{2}=14.43$, $d{f}_{diff}=1$, $p<0.001$) models relative to the 1T2K model, but not relative to each other (${\rm X}_{diff}^{2}=0.33$, $d{f}_{diff}=1$, $p=0.564$). Therefore, the combination of AIC and ${\rm X}^{2}$ goodness-of-fit criteria favors the use of the 2T3K model for ^68^Ga-PSMA-11.

### Parametric evaluation

**Fig. 5:**
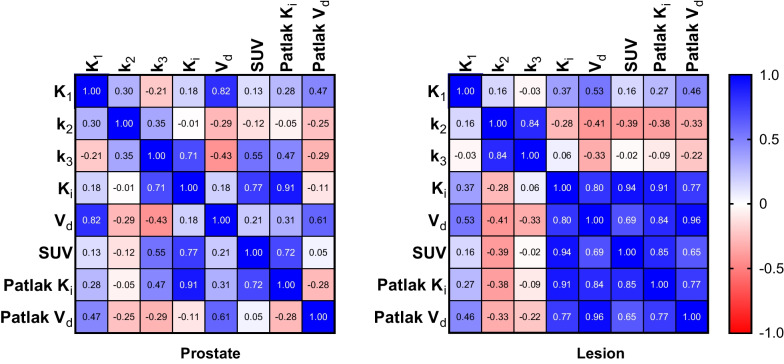
Pearson parameter correlation matrices for reference prostate (*N* = 18) and lesion (*N* = 23) volumes of interest

An assessment of lesion and reference prostate parameter correlations is shown in Fig. 5. Strong correlations were observed between ${K}_{1}$ and ${V}_{d}$ for reference prostate tissue (Pearson *r* = 0.82). Additionally, the net influx rate, ${K}_{i}$, demonstrated a strong correlation (Pearson *r* > 0.7) with SUV in reference prostate and lesions, whether it was calculated by full compartmental analysis or Patlak graphical analysis. Accordingly, the Pearson correlation between the compartmental model ${K}_{i}$ and Patlak graphical model ${K}_{i}$ was 0.91 in reference prostate and lesion. However, there was a moderate positive correlation between the full compartmental model ${V}_{d}$ and the Patlak model *V*_*d*_, especially within the reference prostate (Pearson r = 0.61). In lesions, compartmental model rate constants were uncorrelated or weakly correlated with uptake measures (K_i_, SUV, Patlak K_i_, Pearson |r|< 0.4).

**Fig. 6:**
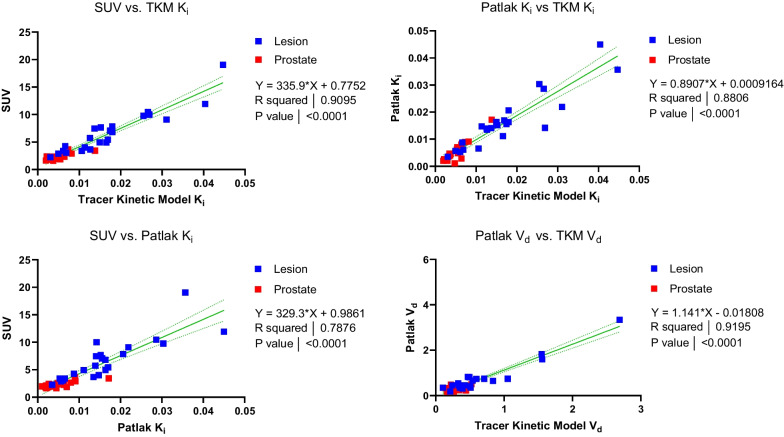
Linear relationships between kinetic parameters, including both reference prostate and lesion volumes of interest. Linear regression results (equation shown) are displayed as a solid green line, with 95% confidence bands in dashed green. *P* values indicate a test of significant non-zero regression slope

**Fig. 7:**
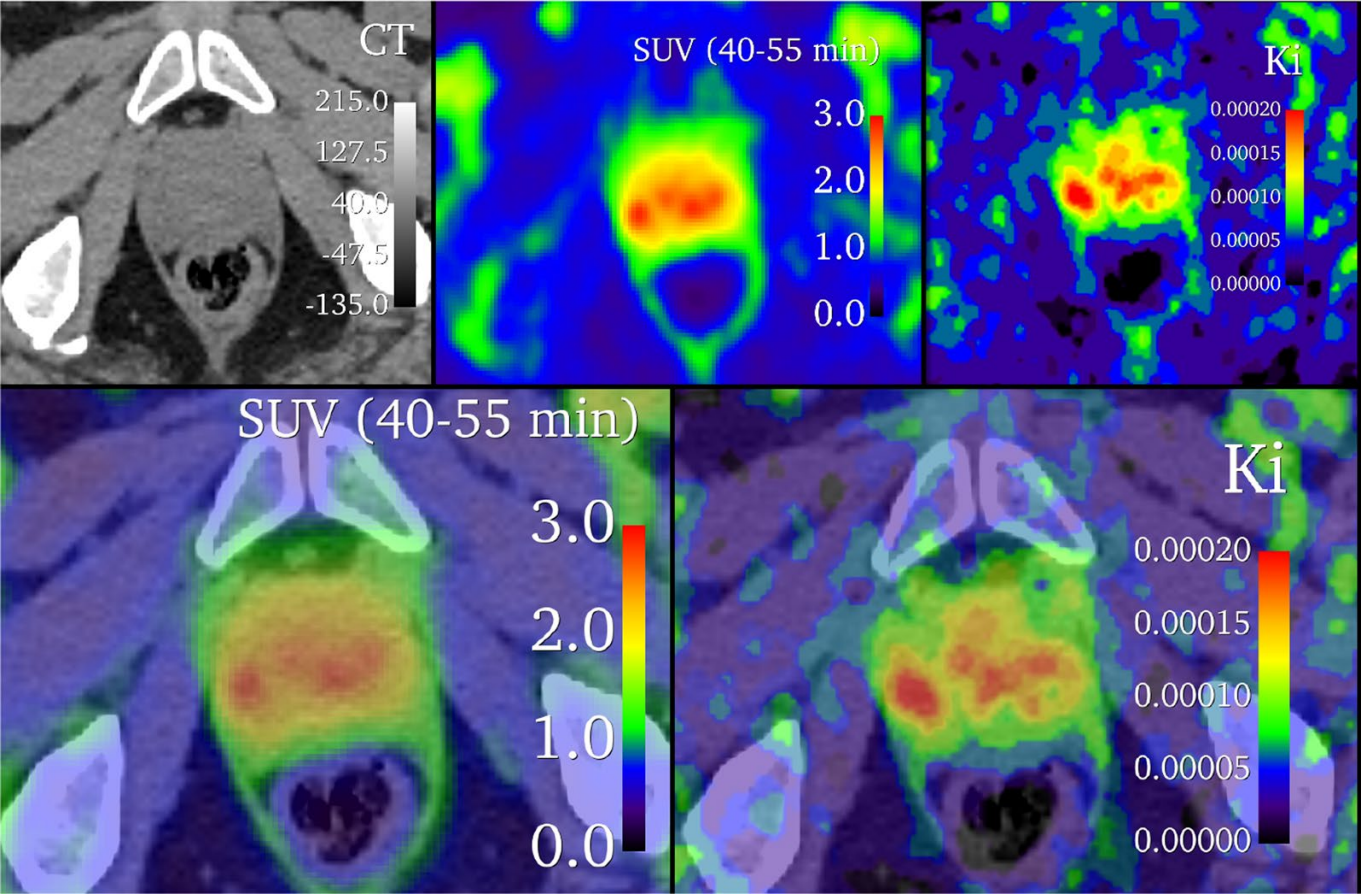
Comparison of SUV and Patlak K_i_^68^Ga-PSMA-11 images. The top row shows parametric images alone, and the bottom row is overlaid with CT at 50% opacity

Linear regressions between SUV, ${K}_{i}$, Patlak ${K}_{i}$, ${V}_{d}$, and Patlak V_d_ are shown in Fig. 6 and Additional file 1: Fig. S1 for combined lesion and prostate, demonstrating large coefficients of determination between SUV, *K*_*i*_, and Patlak *K*_*i*_. However, differential uptake patterns can be observed between SUV and Patlak K_i_ images, as shown in Fig. 7.

SUV and %ID/kg serve as measures of ^68^Ga-PSMA-11 accumulation in static images, but quantification of uptake with %ID/kg yielded less variance than with SUV for both tumor and reference prostate (Table 3). For tumors, mean SUV was 6.7 ± 3.8, while mean %ID/kg was 7.2 ± 3.5. In normal prostate, mean SUV was 2.4 ± 0.6 vs. a mean %ID/kg value of 2.6 ± 0.5. In both cases, the mean %ID/kg is higher than SUV, while the variance in %ID/kg is reduced relative to SUV.

**Table 3:** Median parameter values from 23 lesions in 18 patients

Parameter	Lesion (*n* = 23)	Reference prostate (*n* = 18)
SUV	5.73 [6.74 ± 3.83, 3.84–8.47]	2.34 [2.36 ± 0.59, 1.91–2.65]
%ID/kg	6.64% [7.21 ± 3.47%, 4.77%–8.74%]	2.59% [2.58 ± 0.50%, 2.35%–2.91%]
${K}_{1}$	0.112 [0.123 ± 0.058, 0.087–0.137]	0.089 [0.090 ± 0.034, 0.075–0.104]
${k}_{2}$	0.182 [0.247 ± 0.245, 0.110–0.292]	0.317 [0.317 ± 0.094, 0.254–0.369]
${k}_{3}$	0.032 [0.035 ± 0.029, 0.019–0.038]	0.020 [0.020 ± 0.010, 0.011–0.026]
${K}_{i}$	0.0152 [0.0174 ± 0.0108, 0.0109–0.0217]	0.0052 [0.0052 ± 0.0028, 0.0033–0.0063]
${V}_{d}$	0.471 [0.653 ± 0.580, 0.360–0.652]	0.266 [0.278 ± 0.095, 0.205–0.325]
Patlak ${K}_{i}$	0.0147 [0.0165 ± 0.0102, 0.0099–0.0188]	0.0047 [0.0054 ± 0.0038, 0.0028–0.0070]
Patlak *V*_*d*_	0.458 [0.741 ± 0.688, 0.403–0.742]	0.266 [0.281 ± 0.101, 0.193–0.329]

Shown are comparative median, mean ± standard deviation, first, and third quartile parameter values in lesions and reference prostate tissue. SUV and %ID/kg values are from static images of data from 40 to 55 min post-injection

Shown are median [mean ± standard deviation, first quartile—third quartile] value comparisons for lesion and normal prostate. Semiquantitative values include the standard uptake value (SUV) and percent injected dose per kilogram (%ID/kg). Quantitative parameters include the kinetic two-tissue, three-rate constant model parameters, net influx rate (${K}_{i}$), distribution volume (${V}_{d}$), Patlak model net influx rate (Patlak ${K}_{i}$), and Patlak distribution volume (Patlak *V*_*d*_)

**Fig. 8:**
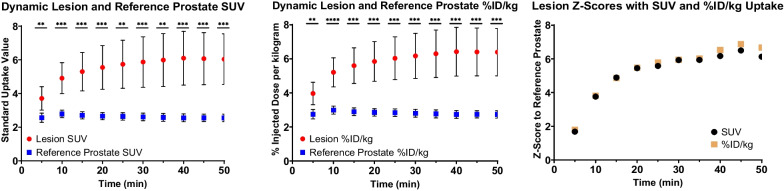
Comparison of standard uptake value (SUV) temporal trends (left) and %ID/kg temporal trends (right) in normal prostate (blue) and lesion (red). Shown is the mean uptake for prostate and lesion at 5 min intervals, calculated as the uptake over a sliding 10-min window centered over each timepoint. Lesion Z-scores (right) are calculated for SUV and %ID/kg using the difference of lesion signal and reference prostate signal, relative to the standard deviation in reference prostate using patient population averages at each timepoint. Error bars represent 95% confidence intervals of the mean. Statistical tests convey the results of a patient-wise paired t test at each timepoint. *p* < 0.01 is indicated by **, *p* < 0.001 is indicated by ***, and *p* < 0.0001 is indicated by ****

Median, mean ± standard deviation, and first and third quartile parameter values are charter for reference prostate tissue and lesions in Table 3. Patient-matched lesion and reference prostate parameter values are displayed in Additional file 1: Fig. S2. Significant differences between parameter values for lesion and reference prostate are noted for K_1_, k_2_, k_3_, K_i_, and SUV in Table 4, while no significant differences in K_i_ and V_d_ estimates were observed between compartmental and Patlak models. Additionally, temporal variations in normal prostate and lesion SUV are compared in Fig. 8, demonstrating a plateau in uptake from 30 to 55 min post-injection. Despite parametric differences between lesion and reference prostate, no significant differences in zone-specific kinetic rate constants or model goodness of fit were observed (Additional file 1: Figs. S3 and S4).

**Table 4:** Comparison of semiquantitative and quantitative parameter values between reference prostate and lesion

Šídák's multiple comparisons test	Summary	Adjusted *P* Value
Reference Prostate K_1_ vs. Lesion K_1_	**	0.0080
Reference Prostate k_2_ vs. Lesion k_2_	**	0.0084
Reference Prostate k_3_ vs. Lesion k_3_	**	0.0080
Reference Prostate K_i_ vs. Lesion K_i_	****	< 0.0001
Reference Prostate V_d_ vs. Lesion V_d_	****	< 0.0001
Reference Prostate SUV vs. Lesion SUV	****	< 0.0001
Reference Prostate Patlak K_i_ vs. Lesion Patlak K_i_	****	< 0.0001
Reference Prostate Patlak V_d_ vs. Lesion Patlak V_d_	****	< 0.0001
Reference Prostate K_i_ vs. Prostate Patlak K_i_	ns	> 0.9999
Reference Prostate V_d_ vs. Prostate Patlak V_d_	ns	> 0.9999

Significance level is set at 0.05 and is corrected for multiple comparisons with the Šídák correction. ns indicates nonsignificant *p* value greater than the significance threshold. *p* < 0.01 is indicated by **, and *p* < 0.0001 is indicated by ****

## Discussion

The results of this study support the use of a two-tissue, three-parameter kinetic model for characterizing the pharmacokinetics of the ^68^Ga-PSMA-11 radiopharmaceutical. ^68^Ga-PSMA-11 exhibits free binding to the extracellular domain of PSMA and slow cellular internalization [21, 22], thus providing a physiological basis for the irreversible two-tissue compartment model and Patlak analysis. Patlak analysis generates estimates of K_i_ and V_d_ through a single linear regression to a subset of late-timepoint data, whereas tracer kinetic modeling uses the full temporal image series to estimate individual kinetic model rate constants. Therefore, tracer kinetic modeling may provide a more robust approach to kinetic analysis when image data are available from the time of tracer injection.

Although the Akaike information criterion suggested that maximal information was preserved by the 1T2k model, chi-square goodness-of-fit criteria suggested that the 1T2k model did not appropriately fit ^68^ Ga-PSMA-11 time-activity curves. Therefore, the 2T3k model is optimal based on dual consideration of the Akaike information criterion and chi-square goodness-of-fit criteria. Previous kinetic evaluations of ^68^ Ga-PSMA-11 for primary prostate cancer have supported the 2T3k or the 2T4k kinetic models, but the findings of this study are consistent with the analysis in high-risk patients established by Ringheim et al. [14, 15, 23]. In comparison with other primary evaluations of ^68^ Ga-PSMA-11 kinetics, the median PSA of patients reported in this study is reduced (6.8 ng/mL) versus Sachpekidis et al. (24.1 ng/mL) and Ringheim et al. (8.64 ng/mL). Additionally, 11/18 patients possessed favorable intermediate grade disease, in comparison with the greater proportion of high-risk disease in other kinetics studies [15, 23].

Kinetic parameters (${K}_{1},{k}_{2},{k}_{3}, {K}_{i}$) exhibited significant differences between lesion and reference prostate tissue, as demonstrated by patient-wise comparison (Fig. 6, Additional file 1: Fig. S2) and statistical comparisons (Table 4). The compartmental model rate constants were also consistent with the ranges reported by Ringheim et al., but reduced net tracer influx (K_i_) and k_3_ in this study likely reflect differences in patient disease severity [15]. Parameter differences between lesion and reference prostate remained significant, regardless of whether ${K}_{i}$ estimates were obtained by full compartmental models or Patlak graphical analysis. ${K}_{i}$ values obtained through compartmental modeling and Patlak analysis were correlated for reference prostate tissue and lesions (Pearson r = 0.91).

Consistent with other reports, lesion ${K}_{i}$ values correlated strongly with SUV for compartmental (Pearson *r* = 0.94) and Patlak (Pearson *r* = 0.85) models, indicating that 40–55 min post-injection SUV metrics provide similar information as ${K}_{i}$ values for lesion detection [15, 23]. Maximal SUVs have also been found to correlate with immunohistochemical PSMA expression and histopathology in patients with prostate cancer [24, 25]. Therefore, it is unlikely that ${K}_{i}$ provides additional information beyond that of either the percentage of the injected dose per kilogram or the measured SUV, simpler methods which are readily implemented in many clinical workflows. Instead, further studies are required to assess if K_i_-based images have utility in imaging of cancers with lower levels of PSMA expression or in the early detection of disease, where improvements in lesion-to-normal tissue contrast, as demonstrated in Fig. 7, may be more impactful toward differentiating lesions from the image background.

Although ${K}_{i}$ (Patlak and full compartmental analysis) correlated strongly with SUV, individual compartment rate constants (K_1_, k_2_, k_3_) demonstrated minimal to slightly negative correlation with SUV. Table 2 and Table 4 demonstrate that K_1_ and k_3_ are significantly elevated in lesions, while k_2_ is significantly decreased. These findings are consistent with increased PSMA expression and PSMA-11 internalization on the prostatic epithelium. The relative reduction in k_2_ in lesions conflicts with previous reported trends [15]. However, the high overall estimation variance across all tracer kinetic parameters likely reflects the heterogeneity of prostate cancer across subjects in this study. The analyzed lesions ranged from Gleason Grade Group 1 to 4, representing a greater focus on intermediate-risk disease than in previous studies, possibly contributing to the observed differences.

Table 3 and Fig. 8 demonstrate that relative to SUV, the %ID/kg has reduced parameter variance. Thus, in quantification of ^68^Ga-PSMA-11 uptake, body mass normalization in the SUV calculation introduces biological noise that reduces diagnostic utility. These observations indicate that ^68^Ga-PSMA-11 uptake quantification as %ID/kg is preferred when using ^68^Ga-PSMA-11 PET uptake thresholds for discrimination of disease. Additionally, reference prostate and lesion uptake plateaus beyond 30 min, with peak lesion-to-reference prostate z-scores occurring at 45 min post-injection. Although discordant with EANM/SNMMI ^68^Ga-PSMA-11 image acquisition guidelines, this finding supports reports indicating that tumor visibility is improved in the 30–45 min window [15, 26–30]. With stable tracer uptake during the 30–55 min window, optimal image acquisition depends largely on count statistics and operational logistics rather than time of acquisition in the first hour post-injection.

As illustrated in Additional file 1: Figs. S3, S4, there were no statistically significant differences between compartmental rate constants, compartmental model ${K}_{i}$, or ${V}_{d}$ between normal prostatic tissue in the central, transitional, and peripheral prostatic zone. Additionally, the chi-square goodness-of-fit criterion was consistent across all prostatic zones, indicating that the model is appropriate regardless of prostatic location. This observation is in contrast to previously reported findings by Pizzuto et al. [31] who reported that ^68^Ga-PSMA-11 accumulation is higher in the central zone than in the transition or peripheral zone. However, the finding was reported during staging for high-risk disease and thus could be attributable as a feature of aggressive disease. In our study, 11% (2/18) patients met or exceeded the average SUV_mean_ reported by Pizzuto et al. in the central zone.

Pelvic ^68^Ga-PSMA-11 dynamic PET scans are challenging to analyze because the iliac arteries are small in caliber compared to larger ventricular blood pools. In this study, a resolution distortion correction method was employed to estimate the artery blood input function based upon the 3-dimensional imaging characteristics of the PET scanner and the local tissue geometry around a segment of the iliac artery. As a retrospective study, arterial blood samples were not available to use as a reference standard to validate the resolution distortion correction method in these patient studies. The resolution distortion correction algorithm was applied to each frame in the dynamic image sequence to generate an arterial blood input function. This method assumed that the background region surrounding the selected vessel segment was homogeneous. Contamination of the background region from small vessel branches or non-homogenous tissues in the proximity of the vessel would result in biased correction factors. For this study, iliac artery segments were carefully selected in each patient to minimize background region contamination from adjacent tissues. Since the vessel and background VOI model were defined on an early phase image, patient motion would potentially impact the accuracy of the estimated correction factors.

This study, although consistent with other literature reports in its findings, has several limitations which should inform its interpretation. The patients enrolled in this study all had biopsy-proven disease, and therefore, no healthy control patients were included in the study. Reference prostate was sampled contralaterally to lesions under the guidance of board-certified physicians with knowledge of post-surgical pathology, minimizing the risk of microscopic disease invasion of control tissue. Despite a limited sample size, the study still provided statistical power to suggest optimal model configuration and kinetic parameter differences between lesion and reference prostate. Additionally, the scope of patients included in this study is primarily limited to intermediate-risk disease, and only patients who were candidates for prostatectomy received ^68^Ga-PSMA-11 PET/CT scans. The study included no low-risk patients, and only a single high-risk patient. Additionally, the demographics of patients meeting the study risk criteria were highly racially homogeneous. The present study was performed on presurgical research scans and did not acquire list-mode data past 55 min. Thus, the kinetics and late time-frame SUV images are temporally constrained and do not fully probe the timeframes recommended by EANM and SNMMI [26].

## Conclusion

The two-tissue compartment model with irreversible binding is appropriate for kinetic analysis in ^68^Ga-PSMA-11 imaging and is applicable to central, transitional, and peripheral prostate, regardless of tumor involvement. Kinetic parameters (K_1_, k_2_, k_3_) are useful for distinguishing prostate cancer lesions from normal prostatic tissue, and kinetic parameters provide information about tissue physiology that is independent from SUV-based metrics and Patlak (K_i_) net influx rate. Assessment of static images with %ID/kg reflects tumor uptake with less intrinsic variance than SUV.

### Supplementary Information

**Additional file 1**. Additional supporting figures. Referenced materials include additional parameter regressions (S1), patient-matched parameter values for lesion and reference prostate (S2), comparison of kinetic parameter values by prostatic zone (S3), and reference prostate chi-square goodness-of-fit values by prostatic zone for the 2T3k kinetic model (S4).

## Data Availability

Data for this publication were acquired in conjunction with NCT04936334. All data and materials presented in this manuscript will be made available upon reasonable request to the corresponding author.

## Abbreviations

PET: Positron emission tomography
PSMA: Prostate-specific membrane antigen
^68^Ga-PSMA-11: ^68^Ga-Glu-NH-CO-Lys-(Ahx)-HBED-CC
SUV: Standard uptake value
NCCN: National Comprehensive Cancer Network
OSEM: Ordered-subsets expectation maximization
PSF: Point-spread function
TOF: Time of flight
CT: Computed tomography
VOI: Volume of interest
ROI: Region of interest
TAC: Time-activity curve
IDIF: Image-derived input function
1T2K: One-tissue, two-parameter
2T3K: Two-tissue, three-parameter
2T4K: Two-tissue, four parameter
AIC: Akaike information criterion
EANM/SNMMI: European Association of Nuclear Medicine/Society of Nuclear Medicine and Molecular Imaging.
Ref.: Reference
