# Supplementary material for: Comparison of tracer kinetic models for 68Ga-PSMA-11 PET in intermediate-risk primary prostate cancer patients
Source: EJNMMI Res. 2024 Jan 10;14:6. doi: 10.1186/s13550-023-01066-2 (PMC10781928; doi:10.1186/s13550-023-01066-2)
Supplement: Supplementary file 1 — Additional file 1. Additional supporting figures. Referenced materials include additional parameter regressions (S1), patient-matched parameter values for lesion and reference prostate (S2), comparison of kinetic parameter values by prostatic zone (S3), and reference prostate chi-square goodness-of-fit values by prostatic zone for the 2T3k kinetic model (S4). [file 13550_2023_1066_MOESM1_ESM.docx]

# Supplementary Materials

Supplemental Figure 1: Multiple single-parameter regressions. Shown for each set of parameters are the linear regression equation, coefficient of determination, and p-value testing for nonzero regression slope. Linear regression line of best fit is displayed as the solid green line, with 95% confidence bands in dashed green.

Supplemental Figure 2: Lesion-wise parameter comparisons in reference prostate tissue (blue) and lesion (red). In patients with multiple lesions, a single reference prostate region was duplicated for comparison with each lesion.

Supplemental Figure 3: Comparison of rate parameters across different regions of the prostate. No statistically significant differences were observed between prostatic zones or between left and right prostatic hemispheres for the parameters shown.

Supplemental Figure 4: Comparison of $X^{2}$ goodness-of-fit values for the 2T3k model time-activity curves. Results of a one-way nested ANOVA indicated that there were no significant differences in model appropriateness between prostatic zones (p=0.081), or between left and right prostate hemispheres. Additionally, pairwise comparisons of each prostatic zone yielded no significant differences using Tukey’s pairwise comparison method (central zone – transitional zone, p=0.5635; central zone – peripheral zone, p=0.3744; transitional zone – peripheral zone, p=0.0651).
